# Distinct Age-Specific Effects on Olfactory Associative Learning in C57BL/6 Substrains

**DOI:** 10.3389/fnbeh.2022.808978

**Published:** 2022-02-02

**Authors:** Hung-Lun Chen, Chien-Fu F. Chen, Han-Bin Huang

**Affiliations:** ^1^Graduate Institute of Life Sciences, National Defense Medical Center, Taipei, Taiwan; ^2^School of Public Health, National Defense Medical Center, Taipei, Taiwan

**Keywords:** C57BL/6, olfactory two-alternative choice task, olfactory associative learning, brain maturation, olfactory habituation/dishabituation test

## Abstract

C57BL/6 is the most widely used mouse strain in the laboratories. Two substrains of C57BL/6, C57BL/6J (B6J), and C57BL/6N (B6N) are well-known backgrounds for genetic modification and have been shown difference in quite a few tests, including open field test, rotarod test, and Morris water maze. However, difference between these two substrains in olfaction-dependent behaviors remains unknown. Here, we used olfactory two-alternative choice task, which is modified to have two training stages, to evaluate animals’ ability in instrumental learning and olfactory association. In the first (rule learning) stage, the mice were trained to use the operant chamber to collect water rewards. An odor cue was provided in the procedure, with no indication about reward locations. In the following (discrimination learning) stage, two odor cues were provided, with each indicating a specific water port. The animals were rewarded upon correct port choices following cue deliveries. We found that during young adulthood (7–10 weeks old), proportionally more B6J than B6N mice were able to pass rule learning (58.3% vs. 29.2%) and ultimately acquire this task (54.2% vs. 25%), with the two substrains showing similar pass rates in discrimination learning (92.9% vs. 85.7%). Surprisingly, at a more mature age (17 weeks old), this substrain difference disappeared. Mature B6N mice had a significant improvement in pass percentages of rule learning and overall task, whereas similar improvement was not observed in the B6J counterparts. Instead, mature B6J mice had an improved speed in rule learning and overall task. We further examined behavioral patterns of 8-week-old B6J and B6N mice in the olfactory habituation or dishabituation test. We observed normal olfactory habituation from subjects of both substrains, with the B6J mice exhibiting stronger investigative responses to newly presented odorants. These results reveal for the first time that B6J and B6N mice are different in acquisition processes of a behavioral task that requires instrumental learning and olfactory association, and that maturation appears to employ different effects on these two substrains during these processes. Furthermore, young adult B6J and B6N mice might be similar in olfactory habituation but different in the olfactory aspects of novelty seeking.

## Introduction

To survive and enhance reproductive success, animals must be able to recognize environmental chemical signals and generate appropriate behavioral responses. In mammals, most external chemical signals are perceived through the olfactory system and are therefore odor molecules. Some odors can instinctively evoke behaviors in animals, which suggests that biological interpretation of an odor can be genetically predetermined (Wyatt, 2014). However, many odor percepts and their meanings are acquired later in life (Cain et al., 1995; Wilson and Stevenson, 2006). How age might affect this lifelong task remains limitedly understood. Current understanding of the age effect on olfactory-associated functions has been mainly from studies of neonatal and aging subjects (Sullivan et al., 2000; Moreno et al., 2014; Kondo et al., 2020). Information regarding olfactory function of more mature adults is lacking. Given that olfactory decline is a salient biomarker that often occurs ahead of major symptoms of numerous neurodegenerative diseases (Doty, 2017), information about olfactory function across adult lifespan is needed for better evaluation of pathological situations that may occur later in life.

C57BL/6 is one of the most widely used mouse strains in biological and biomedical research. Two substrains of C57BL/6, C57BL6/J (B6J), and C57BL6/N (B6N) are common backgrounds for mutant mice (Mekada et al., 2009; Shoji et al., 2016). B6J and B6N mice have been reported to have accumulated dozens of single nucleotide and structural variants across multiple genes (Simon et al., 2013; Lilue et al., 2018) and being different in physical activity, anxiety-like behaviors, motor coordination, balance, spatial learning, and pain sensitivity (Bryant et al., 2008; Matsuo et al., 2010; Simon et al., 2013; Ashworth et al., 2015; Capri et al., 2019). However, difference between B6J and B6N mice in olfaction and olfactory associative learning has not been reported. As olfaction is the predominant sense for the mouse models, it is of importance to understand potential background effects on the performances of behavioral tests that require or are associated with olfactory cues.

In this study, we aimed to investigate age and substrain effects on learning that involves olfactory processing. Using a modified olfactory two-alternative choice task, we examined acquisition processes of B6J and B6N mice in the stepwise training paradigm. This design allows us to evaluate potential effects of age and genetic background on instrumental and olfactory associative learning. The results revealed several substrains and age-specific difference in acquisition of the task and imply that B6J and B6N mice might undergo different functional brain maturation during adulthood.

## Materials and Methods

### Animals

All animal subjects are male mice purchased at the age of 7 weeks. Upon arrival, they were housed in a group of three or four mice per cage with their littermates out of the box. All animals were housed in the same breeding room on a 12-h dark/12-h light cycle (dark from 7 PM to 7 AM). We purchased B6J (C57BL/6JNarl) mice from the National Laboratory Animal Center of Taiwan, which introduced this mouse strain from the Jackson laboratory in 1995. B6N (C57BL/6NCrlBltw) mice were purchased from an authorized local vendor of Charles River laboratory: BioLASCO Taiwan Co., Ltd. (Taipei, Taiwan). For olfactory two-alternative choice task, B6J and B6N mice aged from 7 to 10 weeks old were used as young adult groups. B6J mice in this group (B6J-Y, *n* = 24) are composed of 7-week-old (*n* = 11) and 10-week-old (*n* = 13) mice. The young B6N mice (B6N-Y, *n* = 24) are composed of 8-week-old (*n* = 11) and 9-week-old (*n* = 13) mice. We are aware that many studies view 7-week-old mice as adolescents (Vetreno et al., 2014; Brust et al., 2015; Schulz and Sisk, 2016). By this standard, a portion of B6J-Y mice may start the training in their late adolescent periods. All mice in mature adult groups started the training at 17 weeks old (B6J-M, *n* = 24; B6N-M, *n* = 18). We have a small number of B6N mice (*n* = 5) that began the training at 12 weeks old. To prevent ambiguity and complexity in data interpretation, these animals were not pooled into B6N-M group. For olfactory habituation or dishabituation test, we used 8-week-old B6J (*n* = 5) and B6N (*n* = 6) mice. We trained animals at approximately the same time each day, between 10 AM and 5 PM. All experiments were performed at National Defense Medical Center (NDMC) and approved by Institutional Animal Care and Use Committee of NDMC.

### Training Apparatus and Procedures of Olfactory Two-Alternative Choice Task

**FIGURE 1:**
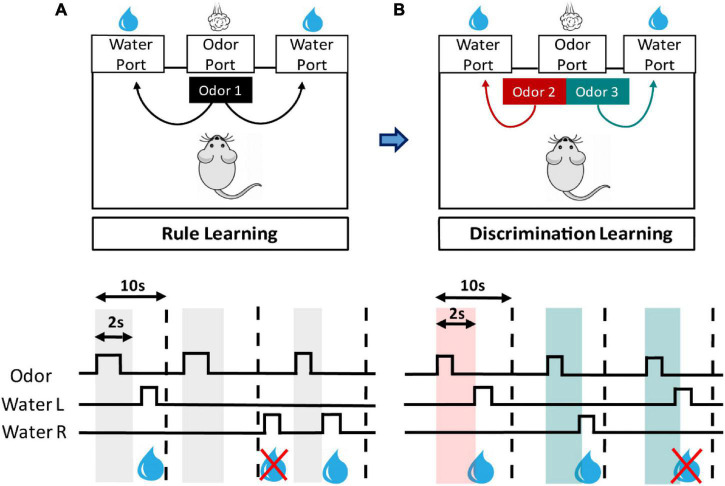
Design of olfactory two-alternative choice task. This task is composed of stepwise training stages for rule learning and discrimination learning. **(A)** In rule learning, the mouse is trained to learn that poking its nose into central (odor) port and subsequently into left or right side (water) port will be rewarded with water. Nose-poke events are detected by an infrared system installed in each port, and signals are continuously recorded by the computer. **(A, lower panel)** A trial begins when the mouse pokes its nose into odor port (square waves of odor trace) and that triggers a 2-s odor cue (gray vertical bars) from the port. If the mouse then pokes into left or right water port (square waves of Water L and R traces) within a 10-s window (vertical dashed lines) will trigger a reward of 15 μl water. **(B)** When reaching the criteria for rule learning, the mice proceed to the training for discrimination learning, in which two odor cues are provided, and each has an associated water port. **(B, lower panel)** In a trial of discrimination learning, two odor cues are randomly given (red and green vertical bars). The mouse has to recognize the cue type and enter the correct water port within the time window to receive a water reward.

The mice were trained in a custom-made operant conditioning chamber (outside dimension: 180 mm (W) × 150 mm (D) × 185 mm (H); inside dimension: 160 mm (W) × 140 mm (D) × 130 mm (H), by Med Associates, Inc., (Vermont, VT, United States) which is controlled by micro 1401 and Spike 2 (Cambridge Electronic Design Ltd., Cambridge, United Kingdom) (Supplementary Figures 1A,B,E). Three ports are mounted onto the same wall of the chamber, with each side port 50 mm away from central port (center to center) (Supplementary Figure 1C). All three ports are equipped with an infrared system to detect port visits (Supplementary Figure 1C). The two side ports are equipped with a water delivery system, which includes a stainless dipper cup with a volume of 15 μl, a water container, and a motorized metal arm that raises the cup to the port in each successful trial (Supplementary Figure 1D). The central port is connected to an odor delivery system. Before training, animals were allowed to explore the operant chamber 10 min a day for 2 consecutive days. During training, animals were able to freely interact with the chamber for 30 min in each day. This daily 30-min training time is defined as a session. All animals were water-deprived throughout training, with daily access to water for 1 h (from 5 to 6 PM). We kept track of their weights to ensure that they were at least 80% of their baseline weights to prevent any welfare concern of the mice (Tucci et al., 2006). Several versions of olfactory two-alternative choice task had been reported previously (Uchida and Mainen, 2003; Frederick et al., 2011; Carlson et al., 2018). In our protocol, we separated the procedures into two major steps: rule learning and discrimination learning (Figure 1). The former is an olfaction-relevant procedural learning, whereas the latter requires odor discrimination skill and correct association between odor cues and the water ports. This design allowed us to evaluate animals’ performance in different learning types.

#### Rule Learning

All animals were first trained to learn the rule that poking its nose into central port and subsequently into one of the side ports will be rewarded with water (Figure 1A). It is innate for mice to poke its nose into holes. A typical trial begins when the mouse’s nose enters central port. The intruding snout blocks infrared beam inside the port and triggers a 2-s *p*-Cymene (C0513, TCI America, Portland, OR, United States) stimulus from the port. If the mouse pokes its nose into either side port within a 10-s window following the initiation of odor delivery, then it will be rewarded with water. Each mouse had one session (30 min) in each day to explore the chamber. We set minimal intertrial interval to be 5 s, and this potentially limited the number of trials in the 30-min session. We often saw that animals have none or sparse water rewards during the initial training days. Some animals may afterward enter a phase where their daily reward numbers abruptly increased and remained high thereafter. We named this initial low-reward phase as exploring phase and the phase with a higher reward number per session as learned phase. The time point, an animal entered learned phase, was judged based on its daily reward number. We considered an animal entering learned phase when it collected at least 10 daily rewards for two consecutive days. The first over-10-reward day is defined as the beginning of learned phase. The mice were given a pass on rule learning when they collected 200 rewards and then proceed to discrimination learning. Generally, mice that could not accumulate 200 rewards by the 15th daily session were considered failing rule learning stage and were removed from the training cohort. However, we gave some individuals extended training if their average rewards in the 14th and 15th sessions were 20 or above.

#### Discrimination Learning

In discrimination learning, two odor cues are provided (limonene and isoamyl acetate, L0047 and A0033, respectively, TCI America), and the mice have to recognize the cue and subsequently enter the associated water port to receive a water reward (Figure 1B). A trial was initiated by the central nose poking behavior that triggers a 2-s odor cue, which was randomly chosen from the two odors. A trial was terminated when the mouse poked into an incorrect port or did not respond to the cue in 10 s. We set right water port associated with limonene and the left port with isoamyl acetate, and this association was applied to all subjects throughout the training. Similarly, mice also had one 30-min session in each day to explore the chamber during the training, with an intertrial interval at least 5 s. A mouse was considered passing this stage of training when it reached 80% correct responses in a session. A mouse failed the training if it could not reach this criterion within 20 sessions (days).

Two operant chambers are installed in a well-ventilated cabinet, with the bottom for rule learning and the top for discrimination learning (Supplementary Figure 1E). We ran one training at a time. The door of the cabinet is closed during training to limit ambient lighting and prevent external visual stimuli (Supplementary Figure 1F).

All used odorants were first dissolved in mineral oil and diluted to vapor pressure at 15.2 ppm. 2 ml of the diluted odorant was then placed into a 10-ml glass vial. An odor stimulus was provided by directing air flow (at 0.6 L/min) into headspace of the odorant vial. Odorant molecules were then mixed with a constant air flow (at 2 L/min) for further dilution. The delivery of odors was controlled by an olfactometer, which was incorporated with the operant chamber (Med Associates, Inc.) and controlled by micro 1401 and Spike 2 (Cambridge Electronic Design Ltd., United Kingdom).

### Olfactory Habituation or Dishabituation Test

Experimental procedure of olfactory habituation or dishabituation test is adapted from two previous studies (Yang and Crawley, 2009; Arbuckle et al., 2015). We conducted the test using a regular laboratory mouse cage with the lid on and the metal frame removed. Before a test started, the animal was placed in the cage and had a 30-min acclimation time, during which an unscented cotton swab was placed at the center of the cage, with the cotton end 4 cm above the bedding material. During a test, in each trial, a cotton swab scented with *p*-Cymene (p), isoamyl acetate (i), or limonene (l) was placed in the cage for 3 min in a sequence: p, p, p, i, i, i, l, l, l, p, p, p. Each trial is separated by 1-min resting time, during which the scented cotton swab was removed from the cage. Animals were placed back to their home cage after test. Behaviors of the tested animal in each trial were recorded and later analyzed. Investigation was defined when the animal approached or sniffed within a radius of 2 cm from the scented cotton.

### Quantification and Statistical Analyses

During a training session of rule and discrimination learning, timings of all events, including a port entrance, an odor cue delivery and a water reward were recorded by Spike 2 for further off-line analysis. The mouse’s performance in rule learning was plotted as a line chart using daily cumulative water reward numbers. Phasic difference in the line in slope was calculated using linear regression. In discrimination learning, the mouse’s learning progress was evaluated by correct rate of each daily session, which is the ratio of the number of collected rewards to the number of trials in a session. Statistical analysis and figure production were performed using OriginPro 8, Microsoft PowerPoint, Microsoft Excel, and Adobe illustrator. For statistical comparisons, in the cases that we compared data of two independent groups, we used Student’s *t-test* or Welch’s *t*-test depending on the result of *F* test. We used paired *t*-test to compare phasic difference in the parameters from the same animal subjects in rule learning. Two-way ANOVA was used to analyze the effects of age and substrain on the length of learned phase of rule learning. One-way ANOVA was used to compare reward numbers collected by different groups before entering learned phase of rule learning. Pearson’s chi-squared test was used to verify whether pass percentages of the two substrains are different in different training stages. Finally, we used generalized estimation equations (Zeger et al., 1988), paired *t*-test and Student’s *t*-test to analyze data of habituation or dishabituation test. All statistical tests are two-tailed, and error bars in the figures are standard errors of the mean.

## Results

### B6J and B6N Mice During Young Adulthood Are Different in Rule (Instrumental) Learning Acquisition

**FIGURE 2:**
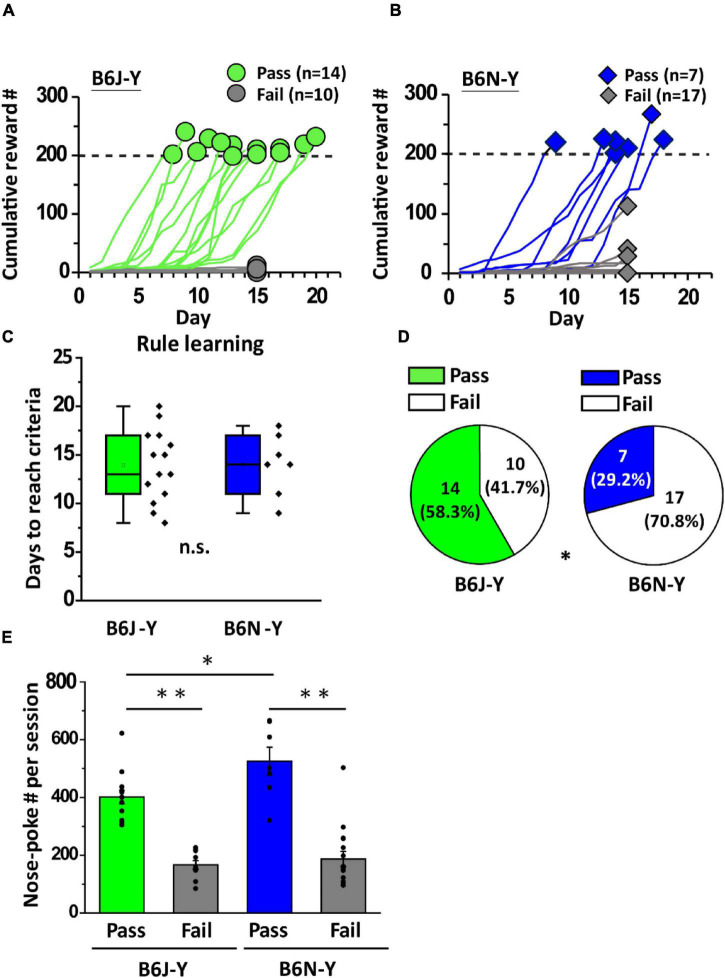
B6J and B6N mice during young adulthood had distinct pass percentages of rule learning. **(A,B)** Curves of cumulative reward number by day of B6J mice (green lines) and B6N mice (blue lines). Green circles and blue rhombuses represent cumulative reward numbers in the last session of rule learning. Dash lines mark the required number of rewards to pass rule learning. **(C)** Box plots of days to reach criteria of the B6J (green) and B6Y (blue) mice. **(D)** Pie charts showing B6J-Y and B6N-Y mice that passed or failed rule learning. **(E)** Average nose-poke number per session of the mice belonging to different substrains and behavioral groups. *p < 0.05. ^**^p < 0.01. n.s., non-significant.

To investigate the effect of substrain on olfactory associative learning, we used olfactory two-alternative choice paradigm to train young adult B6J and B6N mice. This paradigm is composed of rule learning and discrimination learning (Figure 1A). During rule learning, mice had to perform a correct behavioral sequence (a central nose-poke followed by a nose-poke into left or right water port within a 10-s window) to get water in the operant chamber (Figure 1A). Initial water rewards may be collected by accidents. Once the animal accumulated several rewards, correct sequential behaviors should be reinforced. The operant conditioning should ultimately be learned by the animals. To our surprise, only 14 out of 24 B6J-Y mice were able to accumulate 200 rewards and pass rule learning (green lines, Figure 2A). Daily reward numbers of B6J-Y mice that failed to pass remained extremely low throughout the training process (gray lines, Figure 2A). The number of B6N-Y mice that passed rule learning is even smaller (7 out of 24 mice, blue lines, Figure 2B), with the majority (17 out of 24) failed to meet the passing criteria (gray lines, Figure 2B). Although 1 of the failed B6N-Y subjects collected 113 rewards by day 15, it was not qualified for extended training (see section “Materials and Methods”) and was removed from the cohort. The two substrains do not differ in the number of days used to complete the training (B6J-Y: 13.93 ± 0.99 and B6N-Y: 14.00 ± 1.20, Student’s *t*-test, *p* = 0.93; Figure 2C). However, B6J-Y mice are clearly higher in pass percentage than B6N-Y mice (58.3% vs. 29.2%; B6J-Y vs. B6N-Y; chi-squared test, *p* = 0.042; Figure 2D), indicating a significant substrain effect on the rule learning acquisition.

As innate nose-poking behavior is required for triggering water rewards during early stage of rule learning, how frequently an animal performs a nose-poke whereas in the chamber might affect its chance of getting water rewards. We then calculated average nose-poke number per session (nose-poke frequency) for each animal during rule learning. In both B6J-Y and B6N-Y mice, we found that the ones that passed rule learning are higher than the failed ones in average nose-poke frequency (B6J-Y: 401.20 ± 22.62 vs. 166.43 ± 15.09, Student’s *t*-test, *p* < 0.01; B6N-Y: 525.38 ± 48.31 vs. 186.90 ± 26.45, Student’s *t*-test, *p* < 0.01; pass vs. fail; Figure 2E). Interestingly, whereas small in number, B6N-Y mice that passed rule learning are even higher than the B6J-Y counterpart in average nose-poke frequency (Student’s *t*-test, *p* = 0.015; blue bar vs. green bar in Figure 2E), which suggests that these B6N-Y mice might be the most motivated subjects during rule learning.

### Two Phases of Rule Learning Are Characterized by Distinct Daily Reward Number and Reaction Time to Odor Cue

**FIGURE 3:**
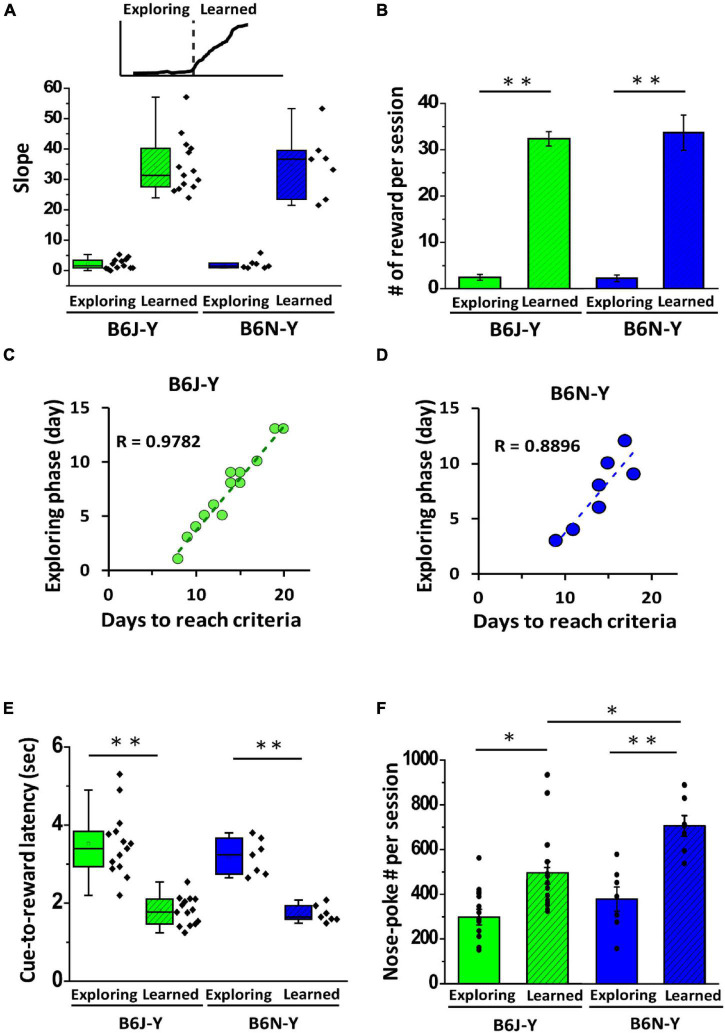
Phasic differences in rule learning of the B6J and B6N mice during young adulthood. **(A)** Individuals learning curves are characterized by distinct slopes during exploring and learned phase of rule learning, as shown in box plots. **(B)** Both B6J-Y and B6N-Y mice were able to collect more water rewards during learned phase of rule learning. **(C,D)** For both B6J-Y and B6N-Y mice groups, the length of rule learning is highly and positively correlated with length of exploring phase. **(E)** Box plots of cue-to-reward latency (time needed to complete a trial) in exploring and learned phases of B6J-Y and B6N-Y groups. A significant reduction in cue-to-reward latency from exploring to learned phase is seen in both animal groups. **(F)** Both B6J-Y and B6N-Y mice exhibited higher nose-poke frequency in learned phase than in exploring phase. *p < 0.05, ^**^p < 0.01.

After further examining learning curves of the mice that passed rule learning (Figures 2A,B), we noticed that these curves, regardless of substrains, are composed of two phases: the initial low-slope phase and the following steep-slope phase. We named these two phases as exploring (low-slope) phase and learned (steep-slope) phase (Figure 3A) (for definition of phases of rule learning, see section “Materials and Methods”). The phasic difference in slope indicates different daily water rewards that these mice received in these two phases. Indeed, both the B6J-Y and B6N-Y mice collected exceptionally more water rewards in learned phase than in exploring phase (B6J-Y: 2.45 ± 0.63 vs. 32.13 ± 1.54, paired *t*-test, *p* < 0.01; B6N-Y: 2.26 ± 0.70 vs. 33.54 ± 3.79, paired *t*-test, *p* < 0.01; exploring phase vs. learned phase, respectively; Figure 3B). These reward numbers in learned phase are reasonable, because each reward is 15 μl of water, and a mouse’s stomach volume is approximately 400 μl (McConnell et al., 2008). Given that the B6J and B6N mice collect over 30 water rewards in a daily session with low spread, it is reasonable to expect that these two substrains could complete learned phase at somewhat similar pace. Indeed, the B6J and B6N mice are not different in days of leaned phase (B6J-Y: 6.43 ± 0.20 days vs. B6N-Y: 6.57 ± 0.57 days, Welch’s *t*-test, *p* = 0.8, Table 1). This result makes us wonder if the diversity in days of rule learning results from diverse exploring phase of the mice. To test this hypothesis, we then analyzed correlation between the number of days in exploring phase and the number of days required to pass rule learning for the B6J-Y and B6N-Y mice. We found that, in both subgroups, the number of days an animal needs to pass rule learning is highly correlated with the number of days it used in exploring phase (*R* = 0.9782 and 0.8896 for the B6J-Y and B6N-Y mice, respectively; Figures 3C,D). To further investigate phasic difference of rule learning, we analyzed the time the mouse needed to complete a successful trial (between the onset of an odor cue and a water reward), designated as cue-to-reward latency. We found that both B6J-Y and B6N-Y subgroups exhibited a significant decrease in cue-to-reward latency in learned phase compared with exploring phase (B6J-Y: 3.52 ± 0.22 s vs. 1.81 ± 0.10 s, paired *t*-test, *p* = 0.00000073; B6N-Y: 3.19 ± 0.17 s vs. 1.72 ± 0.08 s, paired *t*-test, *p* = 0.00018; learned phase vs. exploring phase, respectively; Figure 3E). In other words, the operant conditioning significantly reduced the animals’ response time to an odor cue. In addition, cue-to-reward latency may serve well as a parameter to judge whether a mouse has acquired the rule of collecting water in the operant chamber. We also observed phasic difference in the nose-poke frequency of the animals, as both B6J-Y and B6N-Y mice poked their noses more frequently in learned phase than in exploring phase (B6J-Y: 318.78 ± 30.20 vs. 495.49 ± 50.4, paired *t*-test, *p* = 0.015; B6N-Y: 381.22 ± 54.21 vs. 709.36 ± 46.75, paired *t*-test, *p* < 0.01; exploring phase vs. learned phase, respectively; Figure 3F). These results suggest that these animals, regardless of substrains, were likely more motivated in learned phase of rule learning.

**TABLE 1:** Parameters of four animal groups measured in different training stages and results of statistical comparisons.

	B6J-Y	B6N-Y	B6J-M	B6N-M
**Rule learning**				
Days to reach criteria	13.93 ± 0.99^††^	14.00 ± 1.20	8.78 ± 0.85^††^	10.53 ± 1.39
Exploring phase	7.43 ± 0.96^††^	7.43 ± 1.23	2.08 ± 0.38^††^	4.40 ± 1.11
Learned phase	6.43 ± 0.20	6.57 ± 0.57	6.69 ± 0.64	6.13 ± 0.50
Pass %	58.3*	29.2*^††^	54.0*	83.3*^††^
Nose-poke #/session-Pass	401.20 ± 22.62*	525.38 ± 48.31*	494.73 ± 50.04	559.07 ± 41.55
Exploring phase	318.78 ± 30.20	381.22 ± 54.21	381.07 ± 34.08	422.11 ± 46.63
Learned phase	495.49 ± 50.46*	709.36 ± 46.75*	510.37 ± 58.00	627.30 ± 56.83
Nose-poke #/session-Fail	166.43 ± 15.09	186.90 ± 26.45	157.92 ± 14.69	161.17 ± 37.51
Cue-to-reward latency (s)				
Exploring phase	3.52 ± 0.22	3.19 ± 0.17	3.44 ± 0.33	3.08 ± 0.36
Learned phase	1.81 ± 0.10^†^	1.72 ± 0.08	2.18 ± 0.13^†^	2.08 ± 0.11
**Discrimination learning**				
Days to reach criteria	10.23 ± 1.40	6.17 ± 1.76^†^	8.45 ± 1.69	11.00 ± 1.35^†^
Pass %	92.9	85.7	92.3	73.3
Nose-poke #/session-Pass	474.27 ± 42.90*	1161.69 ± 209.82*^†^	415.23 ± 45.63	558.65 ± 69.67^†^
Nose-poke #/session-Fail	281.45	349.53	499.75	523.28 ± 109.92
**Rule and discrimination learning**				
Days to reach criteria	22.70 ± 0.58^††^	20.17 ± 2.02	17.33 ± 1.77^††^	21.45 ± 2.42
Pass %	54.2*	25.0*^†^	50.0	61.1^†^

*Significantly different between substrains in the same age group, p < 0.05.

*_†_Significantly different between age groups of the same substrain, p < 0.05; _††_ p < 0.01.*

### During Young Adulthood, Proportionally More B6J Than B6N Mice Are Able to Acquire Olfactory Two-Alternative Choice Task

**FIGURE 4:**
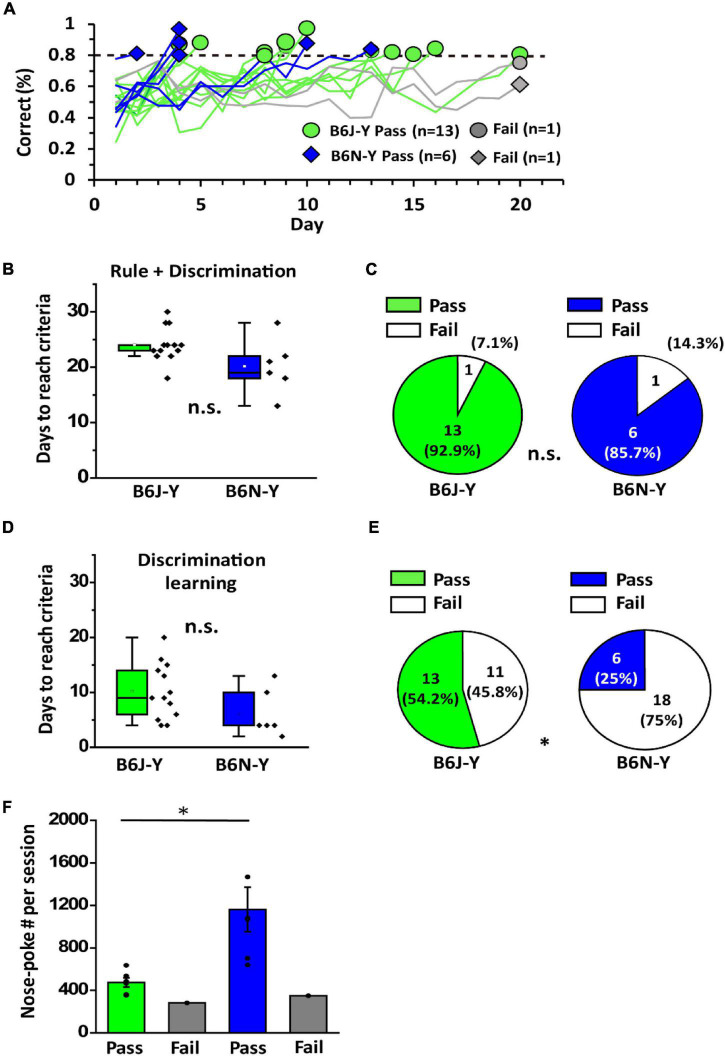
Discrimination learning of B6J and B6N mice during young adulthood. **(A)** Learning curves of the B6J and B6N mice during discrimination learning. **(B)** Days needed for the B6J and B6N mice to reach criteria for passing discrimination learning. **(C)** Pass percentages of the two mice substrains in discrimination learning. **(D)** The numbers of days needed for B6J-Y and B6N-Y mice to accomplish olfactory two-alternative choice task. **(E)** Distinct pass percentages of B6J-Y and B6N-Y mice in olfactory two-alternative choice task. **(F)** The average numbers of nose-poke in a session of B6J-Y and B6N-Y mice that passed or failed discrimination learning. * *p* < 0.05. n.s., non-significant.

The mice that passed rule learning proceeded to discrimination learning paradigm, where they were trained to realize that odor cues of the chamber can be different and that different cues can have different meanings. In this paradigm, each odor cue (isoamyl acetate and limonene) has an associated water port, and a reward is given upon correct water port choice following cue delivery (Figure 1B). We set 80% correct in a single session in 20 days as the passing criteria. Similar to the situation in rule learning, we observed highly diverse learning curves of the animals in discrimination learning (Figure 4A). Overall, the B6J-Y and B6N-Y mice do not differ in days required to pass discrimination learning (10.23 ± 1.40 days vs. 6.17 ± 1.76 days, Student’s *t*-test, *p* = 0.1; Figure 4B). Interestingly, whereas only 29.2% (7 out of 24) B6N-Y mice passed rule leaning (Figure 2D), 85.7% (6 out of 7) of these animals passed discrimination learning (Figure 4C, right). The percentage of the B6J-Y mice that passed discrimination learning is also remarkably high (13 out of 14; 92.9%) (Figure 4C, left). It seems that after passing rule learning, subjects of the two substrains are not different in the ability to establish new olfactory association. When examining total training days for olfactory two-alternative choice task of the mice, we found that the B6J-Y and B6N-Y mice are not significantly different in days to accomplish the whole task (22.70 ± 0.58 vs. 20.17 ± 2.02, Student’s *t*-test, *p* = 0.3; Figure 4D), with proportionally more B6J-Y mice that could acquire the task (54.2% vs. 25.0%; B6J-Y vs. B6N-Y; chi-squared test, *p* = 0.039; Figure 4E). As for nose-poke frequency, in line with the previous result, B6N-Y mice that passed discrimination learning showed higher nose-poke frequency than their B6J-Y counterpart (1161.61 ± 209.82 vs. 474.27 ± 42.90, Welch’s *t*-test, *p* = 0.02; Figure 4F). Therefore, even though only six B6N-Y mice passed discrimination learning, this subgroup appears to have the highest motivation level among the animal subgroups. High motivation level may help them reaching the passing criteria faster, as the majority (4 out of 6) of these B6N-Y mice reached 80% correct within 5 days (Figure 4A).

### During Mature Adulthood, B6J and B6N Mice No Longer Differ in Percentage of Animals That Can Acquire Olfactory Two-Alternative Choice Task

**FIGURE 5:**
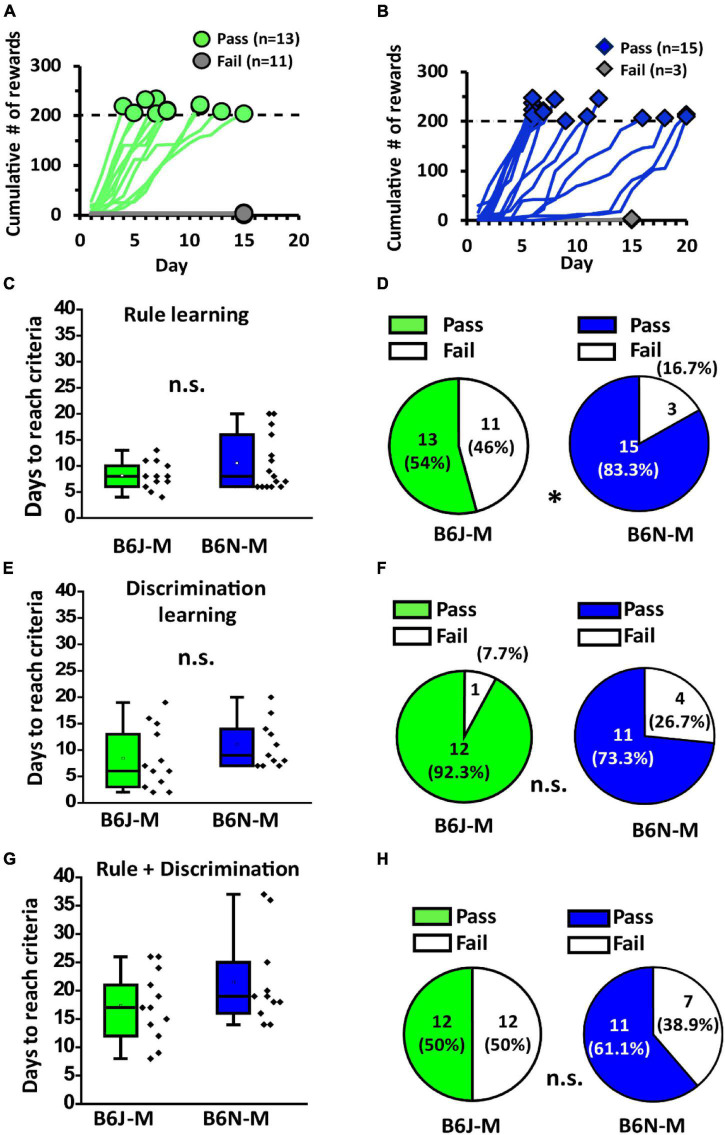
Performance of B6J and B6N mice in training for olfactory two-alternative choice task during mature adulthood. **(A,B)** Learning curves of B6J-M and B6N-M mice in rule learning. **(C)** The numbers of days the B6J and B6N mice used to pass rule learning. **(D)** Pass percentages of B6J and B6N mice in rule learning stage. **(E)** The numbers of days the mice used to pass discrimination learning. **(F)** Pass percentages of the B6J and B6N mice in discrimination learning stage. **(G)** The numbers of days the B6J and B6N mice used to pass the whole task. **(H)** Pass percentages of B6J and B6N mice in olfactory two-alternative choice task during mature adulthood. *p < 0.05. n.s., non-significant.

To investigate age effect on olfactory associative learning of the two mouse substrains, we used the same operant chamber and paradigm to train mature adult B6J and B6N mice (at 17 weeks old, designated as B6J-M and B6N-M, respectively) (Figure 1). Similar to the young adult mice, only a portion of B6J-M and B6N-M mice passed rule learning stage, and their learning curves were also diverse (Figures 5A,B). Likewise, in mature adult mice of both substrains, the ones that passed rule learning exhibited higher nose-poke frequencies than the ones that did not pass (B6J-M: 494.73 ± 50.04 vs. 157.92 ± 14.69, Welch’s *t*-test, *p* < 0.01; B6N-M: 559.07 ± 1.35 vs. 161.17 ± 37.51; Student’s *t*-test, *p* < 0.01; pass vs. fail; Supplementary Figure 2A). However, phasic difference in nose-poke frequency during rule learning was only significant in the B6N-M mice (Supplementary Figure 2B). Like the young adult mice, the two substrains do not differ in days required to accomplish the training (B6J-M: 8.2 ± 7.1 and B6N-M: 10.5 ± 28.8, Welch’s *t*-test, *p* = 0.15; Figure 5C). With all the similarities, however, the two substrains do differ significantly in pass percentage of rule learning (B6J-M: 54% and B6N-M: 83.3%, chi-squared test, *p* = 0.047; Figure 5D), and interestingly, it is the B6N mice with the higher pass percentage during mature adulthood. In discrimination learning, mature adult B6J and B6N mice were not different in days to reach criteria (8.45 ± 1.69 days vs. 11.00 ± 1.35 days; B6J-M vs. B6N-M; Student’s *t*-test, *p* = 0.25; Figure 5E) and pass percentage (92.3% vs. 73.3%; B6J-M vs. B6N-M; chi-squared test, *p* = 0.19; Figure 5F). Unlike the young adult mice, mature adult B6J and B6N mice did not differ in nose-poke frequency during discrimination learning and nor did the pass and fail groups of each substrain (Supplementary Figure 2C). Overall, during mature adulthood, the B6J and B6N mice do not differ in days to accomplish olfactory two-alternative choice task (17.33 ± 1.77 days vs. 21.5 ± 2.42 days; B6J-M vs. B6N-M; Student’s *t*-test, *p* = 0.2; Figure 5G) and pass percentage of the task (50.0% vs. 61.1%; B6J-M vs. B6N-M; chi-squared test, *p* = 0.5; Figure 5H). Performances of the two substrains in olfactory two-alternative choice task, for the most part, are not discriminable during mature adulthood.

### Age-Specific Enhancements and Diminutions Are Observable in Acquisition Processes of Olfactory Two-Alternative Choice Task of Mature Adult B6J and B6N Mice

**FIGURE 6:**
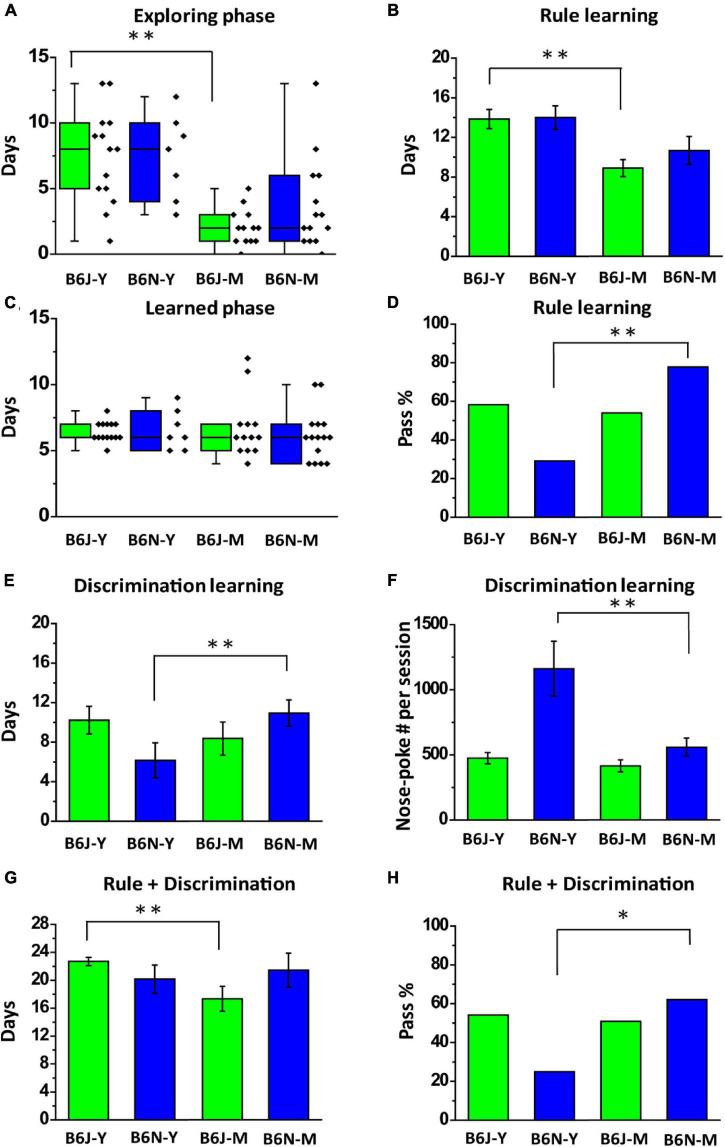
Age-specific effects on B6J and B6N mice in different stages of olfactory two-alternative choice task. **(A)** Box plots of days required for the B6J and B6N mice to complete exploring phase of rule learning. **(B)** The numbers of days the B6J and B6N mice used to pass rule learning. **(C)** Box plots of days required for the B6J and B6N mice to complete learned phase of rule learning. **(D)** Percentages of different age groups of the B6J and B6N mice that are able to pass the stage of rule learning. **(E)** The numbers of days the B6J and B6N mice used to pass discrimination learning. **(F)** Average numbers of nose-poke in a session of the B6J and B6N mice that pass discrimination learning. **(G)** The numbers of days the B6J and B6N mice used to pass the whole task. **(H)** Pass percentages of the whole task that B6J and B6N mice at different age have. *p < 0.05. ^**^p < 0.01.

In addition to substrain effects on the olfactory associative learning, we also investigated age-specific effects on animal behaviors in different training stages of olfactory two-alternative choice task. For example, we found that the mature adult B6J mice spent less days than the young ones did in exploring phase of rule learning (2.08 ± 0.38 days vs. 7.43 ± 0.96 days; B6J-M vs. B6J-Y; Welch’s *t*-test, *p* < 0.01; Figure 6A) and in total training days of rule learning (8.78 ± 0.85 vs. 13.93 ± 0.99, B6J-M vs. B6J-Y; Student’s *t*-test, *p* < 0.01; Figure 6B). This age-specific enhancement was not significant in the B6N mice, even though the time required for rule learning was reduced from 14.00 ± 0.85 days of the B6N-Y to 10.53 ± 1.39 days of the B6N-M (Student’s *t*-test, *p* = 0.13; Figure 6B). Notably, these was no significant age effect on the length of learn phase (Figure 6C, *F*_1_,_47_ = 0.16, *p* = 0.69 for substrain; *F*_1_,_47_ = 0.03, *p* = 0.87 for age). As mentioned before, a significant age-specific enhancement for the B6N mice is in pass percentage of rule learning, which was improved from 29.2% in young adult B6N mice to 83.3% in mature adult B6N mice (Figure 6D). This age effect might be linear, as we observed that 60% (3 out of 5) of 12-week-old B6N mice were able to pass rule learning (Supplementary Figure 3). Conversely, an age-specific diminution in learning speed was observed from the B6N mice during discrimination learning. The B6N-M mice required more days to complete the training compared to the B6N-Y mice (6.17 ± 1.76 days vs. 11.00 ± 1.35 days; B6N-Y vs. B6N-M; Student’s *t*-test, *p* = 0.047; Figure 6E). We also observed that B6N-M mice poke less frequently than the young ones did in discrimination learning (1161.69 ± 209.82 vs. 558.65 ± 69.67; B6N-Y vs. B6N-M; Welch’s *t*-test, *p* = 0.034; Figure 6F). This decrease in nose-poke frequency might be a result of decreased motivation level of the B6N-M mice or alternatively, a result of enhanced efficiency in reward collection of the animals. Overall, mature adult B6-J mice completed the olfactory two-alternative choice task faster than the young adults did (17.33 ± 1.77 days vs. 22.70 ± 0.58 days; B6J-M vs. B6J-Y; Welch’s *t*-test, *p* = 0.015; Figure 6G). But similar age effect was not observed from the B6N mice. Instead, the age effect of the B6N mice is on their pass percentages of the task (25.0% vs. 61.1%; B6J-M vs. B6J-Y; chi-squared test, *p* = 0.018; Figure 6H). But the two B6J age groups do not differ in percentage of the subjects that were able to pass the task (B6J-Y vs. B6J-M; 54.2% vs.50.0%; chi-squared test, *p* = 0.8; Table 1).

### Naïve B6J-Y and B6N-Y Mice Can Both Discriminate Cued Odors but Respond Differently to Novel Scented Objects in the Environment

**FIGURE 7:**
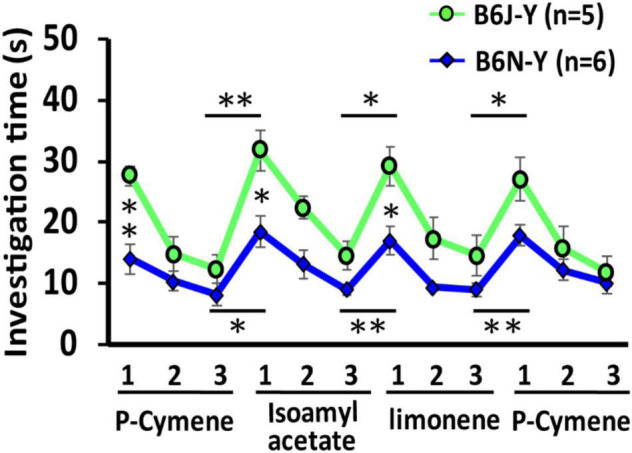
Olfactory habituation or dishabituation test on B6J and B6N mice during young adulthood. A line chart shows how animals of both substrains investigated a cotton swab scented with identical or different odorants in different 3-min trials. *p < 0.05. ^**^p < 0.01.

One untested assumption of this study is that the mice can recognize and discriminate all three odorants used during training. To test this assumption, we used olfactory habituation or dishabituation test with 8-week-old B6J and B6N mice (for details, see section “Materials and Methods”) (Figure 7). Using generalized estimation equations (Zeger et al., 1988), we found that investigation time of B6J-Y and B6N-Y mice over the scented cotton swabs is significantly different (β = –8.51, standard error = 1.41, *p* < 0.001). Furthermore, compared with investigation time during 1st odorant presentation, investigation time during 2nd (β = –8.09, standard error = 1.35, *p* < 0.001) and 3rd (β = –11.4, standard error = 1.52, *p* < 0.001) odorant presentation is significantly decreased, which indicates clear olfactory habituation of the animals to all three odorants. We also observed clear olfactory dishabituation from both substrains, marked by a significant increase in investigation time whenever a new odorant (scented cotton swab) was presented. For B6J-Y mice, olfactory dishabituation was observed in the 1st trial of isoamyl acetate (paired *t*-test, *p* = 0.00071), 1st trial of limonene (paired *t*-test, *p* = 0.038), and 1st trial of the second *p*-Cymene presentation (paired *t*-test, *p* = 0.011) (Figure 7). Likewise, for B6N-Y mice, olfactory dishabituation was observed in the 1st trial of isoamyl acetate (paired *t*-test, *p* = 0.013), 1st trial of limonene (paired *t*-test, *p* = 0.0056), and 1st trial of the second *p*-Cymene presentation (paired *t*-test, *p* < 0.001) (Figure 7). Furthermore, we found that B6J-Y mice responded more strongly than B6N-Y mice in the first trial of odorant presentation (B6J-Y vs. B6N-Y; first *p*-Cymene presentation, *p* = 0.0018; isoamyl acetate, *p* = 0.013; limonene, *p* = 0.016) except for the second *p*-Cymene presentation (*p* = 0.061) (Figure 7). These results together suggest that B6J and B6N mice can discriminate all three odorants without a need of training and reveal a substrain difference in novelty seeking toward the scented objects.

## Discussion

In this study, we investigated difference between two widely used inbred black mouse substrains: B6J and B6N in acquisition processes of an olfaction-dependent behavior and revealed how adulthood ages affect their performances. We used olfactory two-alternative choice task to compare performances of these substrains in olfactory associative learning. In addition, using stepwise training paradigms of this task, we further measured their performances in rule learning and olfactory associative learning. We found that during young adulthood, proportionally more B6J mice were able to learn the task and this difference is attributed to their different pass percentages of rule learning. Interestingly, during mature adulthood, B6J and B6N mice were no longer different in pass percentage of the task. Mature adult B6N mice had an enhancement in pass percentage of rule learning and the overall task, whereas mature B6J mice did not have such improvement. Rather, mature B6J mice had an enhancement in the speed to accomplish the whole task. Finally, using olfactory habituation or dishabituation test, we found that young adult B6J mice exhibited stronger investigation toward novel scented objects compared to their B6N counterparts, suggesting a potential difference of these two substrains in olfactory aspect of novelty seeking in early adulthood.

The observed substrain difference in olfactory two-alternative choice task might be a result of genetic difference between B6J and B6N mice (Mekada et al., 2009; Zurita et al., 2011; Simon et al., 2013; Lilue et al., 2018). Simon et al. have validated 49 variants between B6J and B6N mice, which include 34 coding single-nucleotide polymorphisms (SNPs) and 15 structural variants (SVs). Among these variants, five SNP-affected genes (*Crb1*, *Pdzk1*, *Pmch*, *Adcy5*, and *Nlrp12*) and three SV-containing genes (*Chl1*, *Rpto*r, and *Nnt*) have phenotypic annotations. Some of them are associated with neurological functions of the animal. For example, *Chl1* knockout has been shown related to impaired attention (Pratte et al., 2003), novelty detection (Pratte and Jamon, 2009), and spatial working memory (Buhusi et al., 2013) in mice. Furthermore, mice with *Adcy5* knockout display Parkinsonian-like motor dysfunction (Iwamoto et al., 2003). Though that B6J mice carry an intronic long interspersed element insertion in *Chl1* locus (Simon et al., 2013), their olfactory novelty seeking is stronger compared to B6N mice (Figure 7). Whereas B6N mice carry a private missense variant in *Adcy5*, we observed any motor abnormality from these animals during the training. Therefore, with numerous genetic differences between B6J and B6N mice being identified, how these differences might contribute to their difference in the olfactory two-alternative task remains unclear and requires further investigation.

Age is another factor that has been shown affecting olfactory-related functions of the mice. For example, Patel and Larson showed that 24-month-old mice require more training sessions than 4-month-old mice to complete the task aiming to discriminate 8 different odors (Patel and Larson, 2009). Mice aged 18 months have also been shown inferior to 2-month-old mice in olfactory perceptual learning and odor discrimination capability (Moreno et al., 2014). In contrast to all these findings, which described age-related diminution in olfactory functions, we observed several substrain- and age-specific enhancements in acquisition process of the olfactory two-alternative choice task (Figure 6). These results indicate a possibility that B6J and B6N mice might undergo different functional brain maturation during early adulthood.

Perhaps, the most unexpected result of this study is low pass percentages of the mice in rule learning, which is by design a two-alternative free choice task – a choice of either side port after cue delivery leads to a reward (Trevino et al., 2020). This design may sound easy but turns out to be rather difficult for the animals. There are a number of reasons that potentially make rule learning difficult to pass. First, in our operant chamber, the animal needs to nose-poke into different ports in a specific sequence to receive a reward, whereas in other olfactory learning protocols, such as go/no go (Roesch et al., 2007; Slotnick, 2007; Frederick et al., 2011), Y-maze (Sato et al., 2015), and odor-cued taste avoidance (Slotnick and Coppola, 2015), rewards are provided following single nose-poke or approach to the cue. In addition, the location for reward delivery is often in close proximity to the sampling area, potentially making associative learning easier. Second, to reduce difficulty, stepwise training is commonly applied so that one specific behavior is reinforced at a time (Slotnick, 2007; Frederick et al., 2011). However, in rule learning of our protocol, the animals received no further instruction about the water-giving nose-poke sequence and had to discover the rule by themselves. Another major difference between our and other protocols is the regime of water deprivation. We allowed daily 1-h unlimited water access, whereas other groups have much stricter water-deprivation regime, such as 10-to 30-min daily water access (Roesch et al., 2007), 1–2 ml of water per day (Slotnick, 2007), and 1–3 ml daily water along with 30- to 60-s water access after test (Sato et al., 2015). Thus, we expect that our animals were less dehydrated compared to water restricted animals of other studies. However, it is still unclear how dehydration level would affect motivation of the animal during rule learning, especially when pure nose-pokes in the operant chamber do not lead to a reward? Because body weight drop of the animal is caused by water deprivation, we then generated scatter plots to reveal relationships between body weight ratio (current body weight over baseline body weight) and nose-poke frequency of the animal in a session (Supplementary Figures 4A1–A8). We found that nose-poke frequency and body weight ratio are positively and weakly correlated in the animal groups, regardless of substrain, age, and training result. Therefore, dehydration level of the animals might not be a major factor that affects nose-poke frequency of the animals.

An intriguing question raised from the result is why only a portion of the animals can pass rule learning? One hypothesis is that the higher nose-poke frequency of an animal, the higher its chance to collect a reward by accident and to subsequently reach rule understanding. The failure might be attributed to low nose-poke frequency during training. To determine whether higher nose-poke frequency is correlated with higher reward numbers, we generated scatter plots for four animal groups to reveal relationships between average nose-poke frequency and average reward number during exploring phase (Supplementary Figures 5A1–A4). Given that reward collection patterns were similar once the animals are in learned phase, we only analyze the data during exploring phase. For animals that passed rule learning, we found that there is a positive and strong correlation between nose-poke frequency and reward collection in all the animal groups except B6J-Y (B6J-Y, *R* = 0.6840; B6N-Y, *R* = 0.7928; B6J-M, 0.9275; B6N-M, 0.7740). For the ones that failed to pass, nose-poke frequency and reward collection are moderately correlated in young animals of both substrains (B6J-Y, *R* = 0.5778; B6N-Y, *R* = 0.5323), weakly correlated in B6J-M mice (*R* = 0.4076), and strongly correlated in B6N-M mice (*R* = 0.8767). Therefore, these results support the hypothesis that the higher the nose-poke frequency of an animal the more rewards it may collect during exploring phase, whether or not it passes rule learning.

The second part of the hypothesis is the higher the reward number, the better the chance of an animal to reach rule understanding. Whereas we cannot directly test this hypothesis, we analyzed reward numbers of the animals before entering learned phase. We found that these numbers are ranging from 15 to 59 for B6J-Y mice, 17 to 62 for B6N-Y mice, 25 to 89 for B6J-M mice, and 17 to 68 for B6N-M mice, with the mean 34.50 for B6J-Y mice, 40.00 for B6N-Y mice, 39.15 for B6J-M mice, and 42.53 for B6N-M mice (Supplementary Figure 5B). Group means of the required reward numbers are not significantly different from one another (ANOVA, *F*_3_,_48_ = 0.5534, *p* = 0.6485). This result suggests that the mice of all groups require similar amount of experience to reach rule understanding, regardless of substrain and the adulthood ages. The mice that failed to pass generally collected fewer rewards than the pass animals (Supplementary Figure 5B), with a small fraction of the young mice collecting more rewards than the group minimum (2/10 of B6J-Y mice and 4/17 of B6N-Y mice) or even group average (2/17 of B6N-Y mice) (Supplementary Figure 5B). On the contrary, all mature mice that failed to pass collected fewer rewards than their group minimums. Whereas a testable hypothesis that explains mechanisms underlying the failed cases is not available, we can at least identify two phenotypes of these animals. One feature is the inability to induce water rewards in the operant chamber (reward induction). The other is the inability to transform reward experience into rule understanding (rule generation). We found that most failed cases (B6J-Y: 8/10, B6N-Y: 15/17, B6J-M: 11/11, B6N-M: 3/3) had difficulty in reward induction. Only a small portion of the young mice had problems in rule generation. These results suggest that brain maturation might generally improve rule generation of the mice and that the effect of maturation on reward induction might be substrain-specific. Given that understanding of functional brain maturation during adulthood is still limited (Tian and Ma, 2017), more knowledge is required to further identify neural circuits and mechanisms underlying this substrain- and age-specific changes in rule acquisition.

## Data Availability Statement

The original contributions presented in the study are included in the article/Supplementary Material, further inquiries can be directed to the corresponding author/s.

## Ethics Statement

The animal study was reviewed and approved by Institutional Animal Care and Use Committee of National Defense Medical Center.

## Author Contributions

C-FC and H-LC designed the research and wrote the manuscript. H-LC performed the experiments. H-LC, C-FC, and H-BH analyzed the data. All the authors contributed to the article and approved the submitted version.

## Conflict of Interest

The authors declare that the research was conducted in the absence of any commercial or financial relationships that could be construed as a potential conflict of interest.

## Publisher’s Note

All claims expressed in this article are solely those of the authors and do not necessarily represent those of their affiliated organizations, or those of the publisher, the editors and the reviewers. Any product that may be evaluated in this article, or claim that may be made by its manufacturer, is not guaranteed or endorsed by the publisher.
