# Supplementary material for: Distinct Age-Specific Effects on Olfactory Associative Learning in C57BL/6 Substrains
Source: Front Behav Neurosci. 2022 Feb 2;16:808978. doi: 10.3389/fnbeh.2022.808978 (PMC8847720; doi:10.3389/fnbeh.2022.808978)
Supplement: Supplementary file 1 [file Data_Sheet_1.docx]

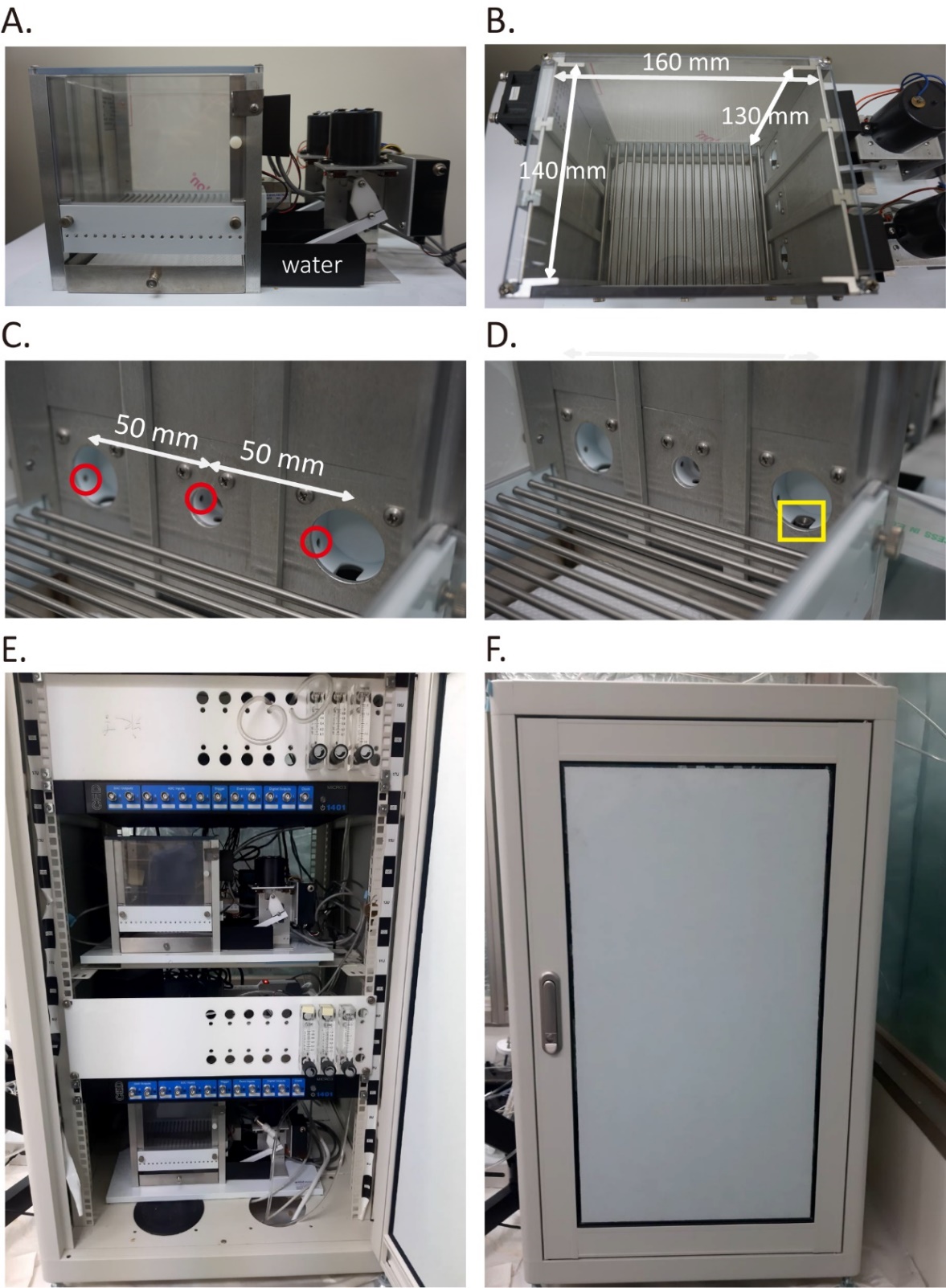
**Supplementary Figure 1. Training apparatus for the olfactory two-alternative choice task. (A)** A side view of the operant chamber. **(B)** A top view of the operant chamber. **(C)** Three ports are located on the bottom of the side wall; the odor (central) port is flanked by two water ports. Each port has an infrared beam detection system indicated by red circles **(D)** A water reward is provided with a stainless dipper cup (yellow rectangle) that is programed to be raised to the port for 1 sec whenever the animal makes a correct choice. **(E)** Two sets of the operant chamber and controlling interface are installed in a cabinet for rule learning (bottom) and discrimination learning (top). **(F)** During training, the door of the cabinet should remain closed.

**S**
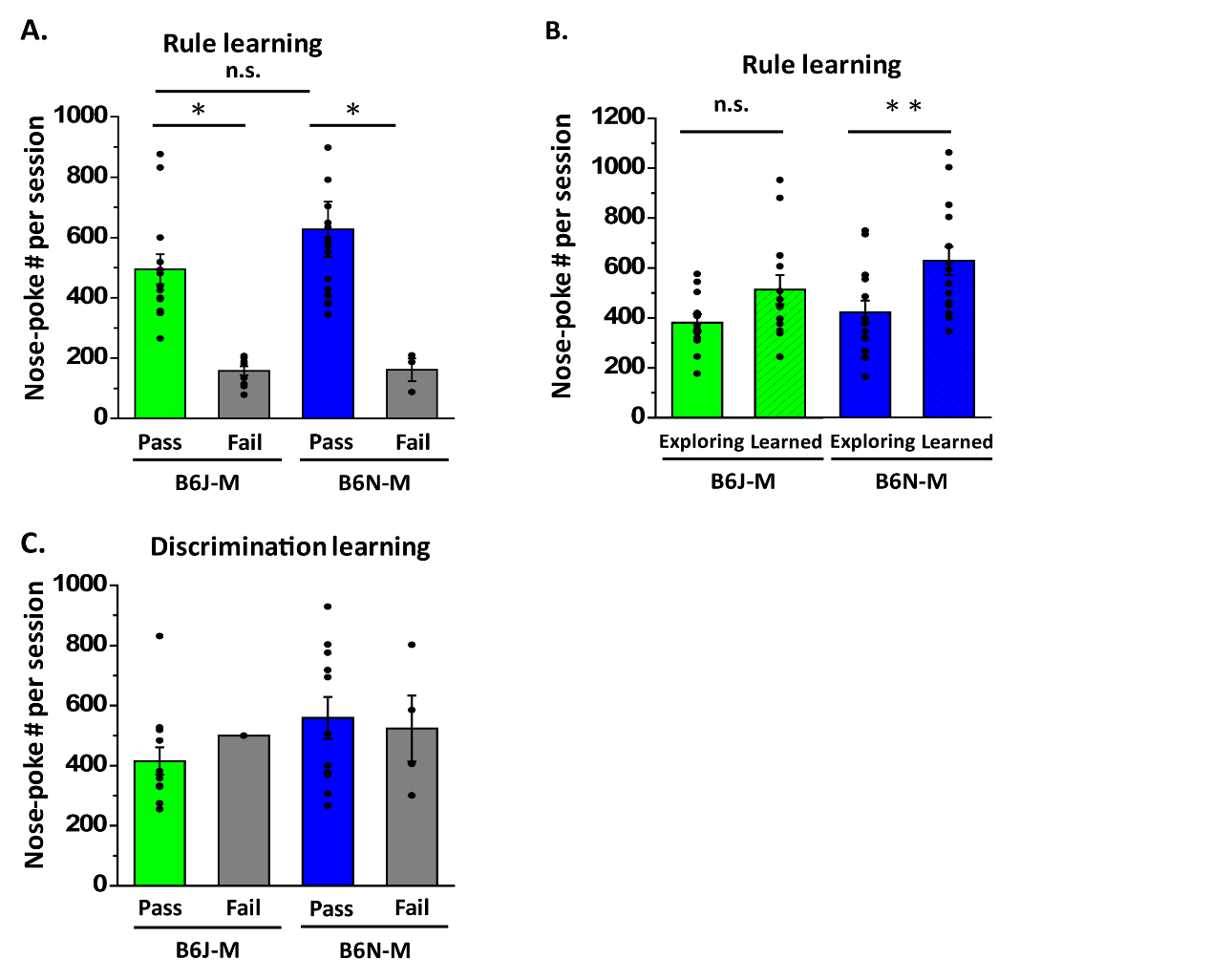
**upplementary Figure 2.** **Nose-poke frequencies of mature adult B6J and B6N mice at different training stages.** **(A)** Nose-poke numbers per session of different groups during rule learning. **(B)** Nose-poke frequencies during exploring and learned phases of rule learning. **(C)** Nose-poke numbers per session of different groups during discrimination learning. ***** *p* < 0.05. ****** *p* < 0.01. **n.s.**, non-significant.


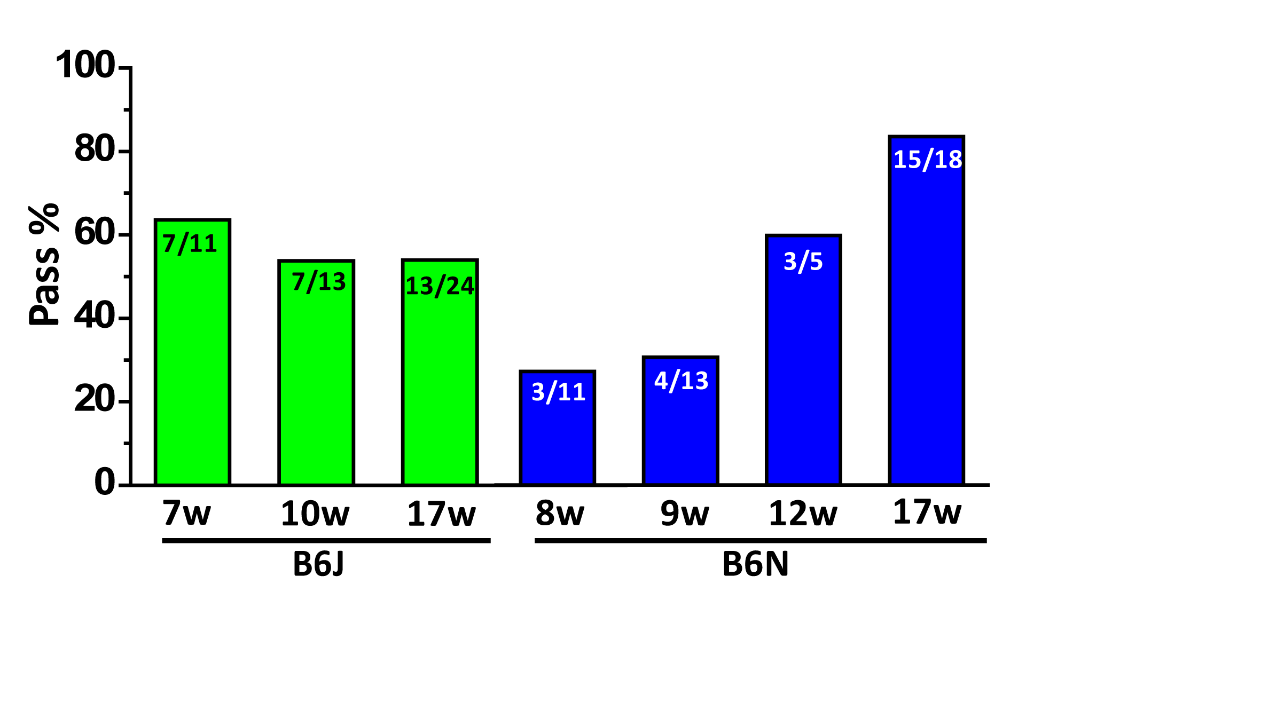


**Supplementary Figure 3. Pass percentages of rule learning stage for B6J and B6Y mice at different ages.**

**
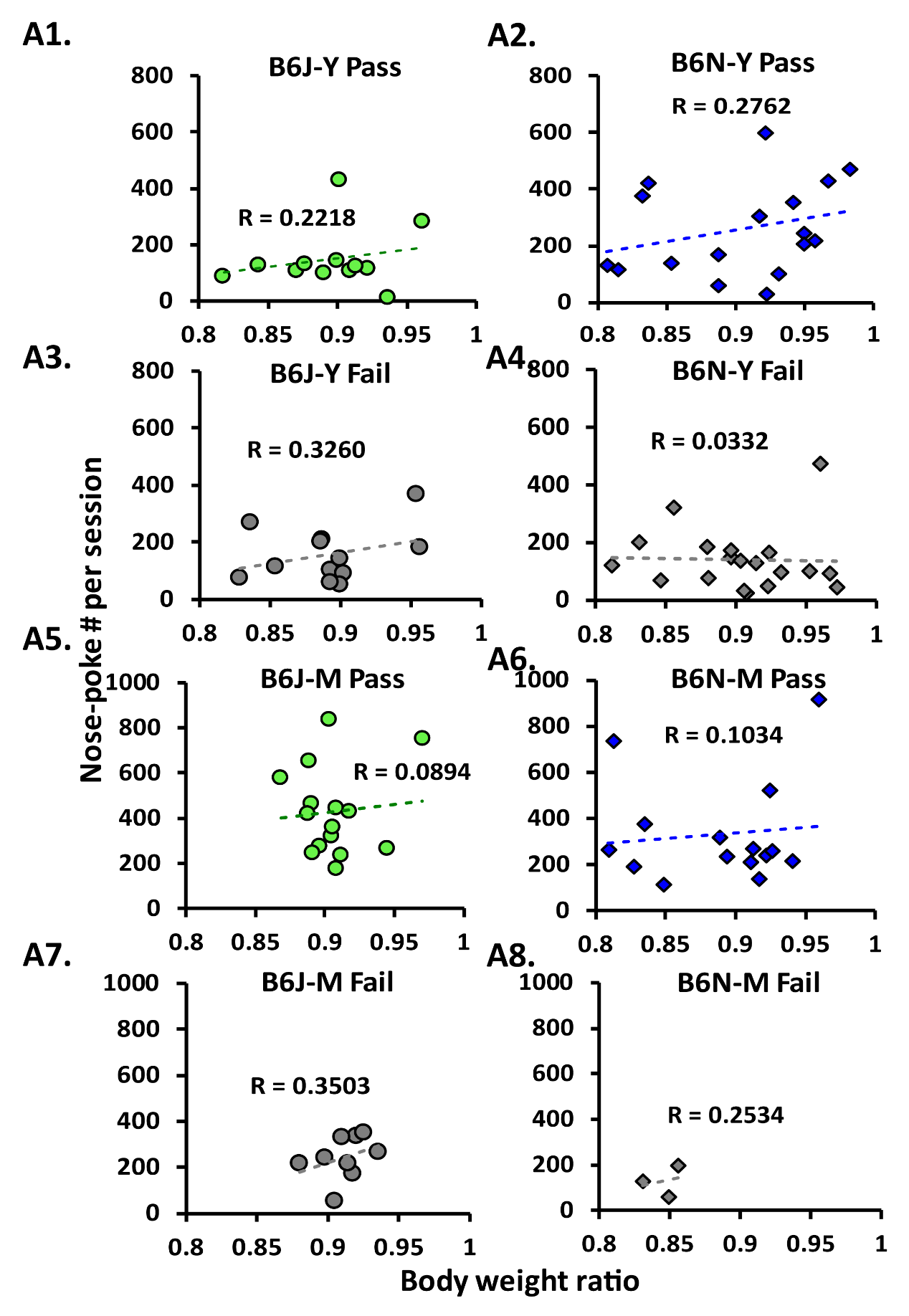
**

**Supplementary Figure 4. Relationships between nose-poke frequency and body weight ratio (current body weight over baseline body weight) for different animal groups.**


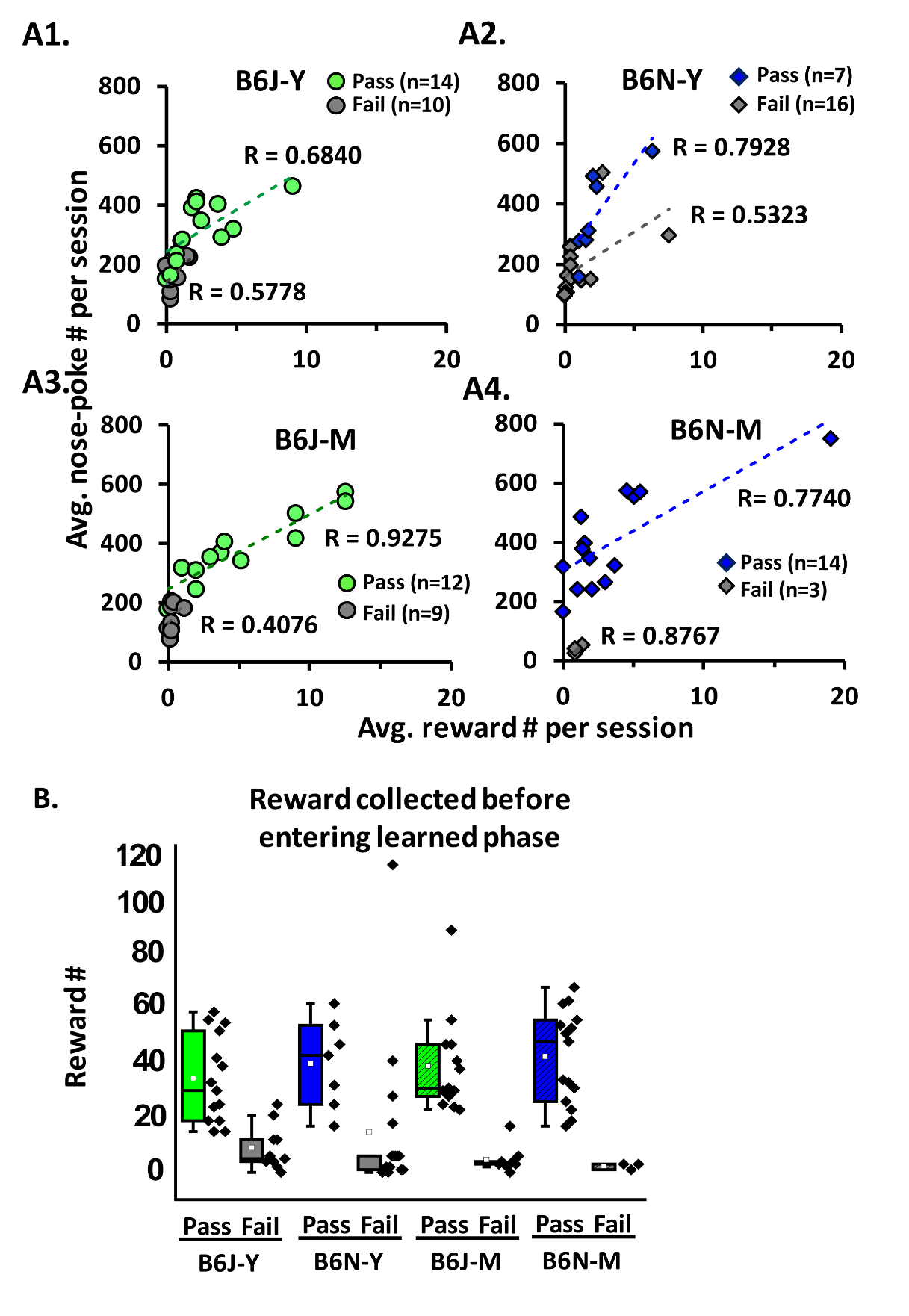


**Supplementary Figure 5. Relationships between nose-poke frequency and reward collection in the exploring phase of rule learning. (A1 to A4)** Scatter plots show relationships between average nose-poke frequency and average reward collection of B6J-Y, B6N-Y, B6J-M, and B6N-M mice. **(B)** Number of rewards collected by each animal before entering the learned phase of rule learning.
